# Ultrasound-Guided Minimally Invasive Tenotomy Utilizing TenJet for Recalcitrant Gluteal Tendinopathy: A Six-Month Interim Analysis From a Prospective Multicenter Study

**DOI:** 10.7759/cureus.115717

**Published:** 2026-09-03

**Authors:** Michael Dakkak, Gabriel Furey, Edward Rosero, David Berkoff, Eugene Y Roh, Sean Bradley, Philip Motley, Geoffrey Hogan, Christopher Varacallo, Jason Genin, Vikas Patel, Christopher J Mehallo

**Affiliations:** 1 Non-operative Sports Medicine, Cleveland Clinic Florida, Parkland, USA; 2 Research and Development, Rothman Orthopaedics, Philadelphia, USA; 3 Non-operative Sports Medicine, Rothman Orthopaedics, Philadelphia, USA; 4 Orthopedic Surgery, University of North Carolina School of Medicine, Chapel Hill, USA; 5 Sports Medicine, Stanford University, Redwood City, USA; 6 Orthopedic Sports Medicine, Atrium Health, Charlotte, USA; 7 Orthopedic Sports Medicine, Rothman Orthopaedics, Philadelphia, USA; 8 Orthopedic Sports Medicine, Ochsner Health, New Orleans, USA; 9 Orthopedic Sports Medicine, Penn Highlands Healthcare, Dubois, USA; 10 Orthopedic Surgery, Cleveland Clinic Foundation, Cleveland, USA; 11 Non-operative Sports Medicine, Cleveland Clinic Foundation, Cleveland, USA

**Keywords:** gluteal tendinopathy, greater trochanteric pain syndrome, lateral hip pain, minimally invasive tenotomy, tenjet, ultrasound-guided tenotomy

## Abstract

Background: Chronic gluteal tendinopathy is characterized by failed tendon healing, collagen disorganization, and breakdown in tendon structure. Traditional conservative management involves nonsteroidal anti-inflammatory drugs (NSAIDs), physical therapy, and corticosteroid injections. However, next steps for recalcitrant cases are lacking consensus. The TenJet device (HydroCision, Inc., North Billerica, Massachusetts, United States), utilizing high-pressure saline to resect degenerative tissue, offers a minimally invasive option to treat gluteal tendinopathy.

Methods: From 2022 to 2025, 46 patients with gluteal tendinopathy refractory to conservative treatment were enrolled across multiple centers with planned 24-month follow-up. Visual Analog Scale (VAS) pain score and Victorian Institute of Sport Assessment-Gluteal Questionnaire (VISA-G) patient-reported outcomes were prospectively collected via REDCap alongside demographics, treatment history, procedural details, and adverse events.

Results: The cohort was predominantly female (44/46, 95.6%), with a mean age of 60.7±8.44 years and a mean BMI of 26.9±4.73 kg/m². The mean VAS improved from 5.81±2.02 at baseline to 3.14±2.24 at two weeks (p<0.001), 2.40±2.00 at six weeks (p<0.001), 2.17±1.87 at three months (p<0.001), and 2.10±2.10 at six months (p<0.001). VISA-G improved from 44.9±14.3 at baseline to 49.0±19.1 at two weeks (p=0.807), 62.2±15.8 at six weeks (p<0.001), 66.9±15.2 at three months (p<0.001), and 69.6±12.6 at six months (p<0.001). One patient developed an adhesive-related rash; no device malfunctions or serious complications were observed.

Conclusions: Ultrasound-guided minimally invasive tenotomy with TenJet was associated with significant, sustained reductions in pain and improvements in function through the six-month follow-up, with a favorable short-term safety profile for chronic gluteal tendinopathy refractory to conservative management.

## Introduction

Greater trochanteric pain syndrome (GTPS) is characterized by lateral hip pain along the greater trochanter, encompassing a spectrum of diverse etiologies [1-4]. The prevalence of GTPS among adults ranges from 10% to 25%, with markedly higher rates observed in women, particularly in middle-aged and older populations [5-9]. Gluteal medius and gluteal minimus tendinopathy are the most predominant cause of GTPS [3,8]. There is a range of pathologies including bursitis, tendinitis, tendinosis, and tendon tears. Tendinosis is differentiated from inflammatory etiologies in that it represents a chronic degenerative process caused by repetitive strain marked by failed tendon healing and disordered collagen remodeling [8,9].

Initial management of gluteal tendinopathy is often focused on conservative measures such as patient education, activity modifications, analgesic medications, physical therapy, and corticosteroid injections for treating inflammation and providing symptom relief [1,4,10-13]. If symptoms are refractory, advanced imaging with either ultrasound or magnetic resonance imaging (MRI) is usually obtained to better characterize the tendon. Further management strategies in recalcitrant cases include platelet-rich plasma (PRP) injections, extracorporeal shockwave therapy, and ultrasound-guided minimally invasive tenotomy [8,9,13-17]. Despite these approaches, a subset of patients fail to achieve substantial pain and functional improvement and are often considered for surgical intervention.

The TenJet device (HydroCision, Inc., North Billerica, Massachusetts, United States) utilizes a dual-lumen 12-gauge needle (2.77 mm in diameter by 7.62 cm in length) to deliver a high-velocity saline jet to enable a minimally invasive approach to the resection of the degenerative tendinopathic tissue. The saline jet, reaching up to 14,000 pounds per square inch of pressure, enables the targeted resection of the diseased tendon while sparing the healthy tissue, a feature made possible by the Venturi effect and a patented needle tip design. TenJet received Food and Drug Administration (FDA) clearance in 2019, and early studies have documented its use in other anatomic locations, including the elbow and medial collateral ligament. Thus, prior clinical evidence regarding TenJet has primarily been derived from non-gluteal applications. To the authors' knowledge, no prior study has specifically evaluated TenJet for gluteal tendinopathy.

Beginning in 2022, an investigator-led, multicenter, prospective cohort patient registry began collecting real-world evidence for ultrasound-guided tenotomy procedures utilizing TenJet for treating elbow, hip, and calcific shoulder tendinopathy. The primary goal of this six-month interim analysis is to report on changes in pain, patient-reported function, and safety outcomes following treatment with TenJet for recalcitrant gluteal tendinopathy.

## Materials and methods

Study design and patient enrollment

The study was conducted at the following hospital/surgery center locations: Stanford Medicine Outpatient Center, San Francisco, California; Avon Hospital, Cleveland, Ohio; New Britain Surgery Center, Chalfont, Pennsylvania; Jefferson Surgery Center at the Navy Yard, Philadelphia, Pennsylvania; Coral Springs Family Health and Surgery Center, Coral Springs, Florida; and Penn Highlands DuBois Surgery Center, DuBois, Pennsylvania. Procedures at Ochsner Healthcare and UNC Healthcare were performed in clinic procedure rooms. This registry was approved by the Western Copernicus Group (WCG) Institutional Review Board (IRB) (approval number: 20215602; sponsor protocol ID: HydroCision 2021-1), with additional site-specific approvals obtained at participating institutions. Seven centers enrolled patients in the protocol "Prospective Multi-center Patient Registry for Patients With Elbow, Hip, and Calcific Shoulder Tendinopathy Treated Utilizing the TenJet System" (ClinicalTrials.gov ID: NCT05352139): Rothman Orthopaedics, Stanford Healthcare, UNC Healthcare, Cleveland Clinic Ohio, Cleveland Clinic Florida, Ochsner Healthcare, and Penn Highlands Healthcare.

While the protocol seeks to enroll patients experiencing tendinopathy from three distinct anatomic locations, the focus of this report is limited to patients presenting with gluteal (hip) tendinopathy. For this analysis, recalcitrant gluteal tendinopathy was defined as lateral hip pain for greater than three months with clinical and MRI or sonographic evidence of gluteal tendinopathy despite at least three months of nonoperative treatment. The registry is ongoing with planned follow-up through 24 months. The present study represents the six-month interim analysis of the gluteal tendinopathy cohort. The protocol's inclusion and exclusion criteria specific to gluteal tendinopathy are shown in Table 1.

**Table 1:** Inclusion/exclusion criteria specific to hip tendinopathy BMI: body mass index; MRI: magnetic resonance imaging; PRP: platelet-rich plasma; CRPS: complex regional pain syndrome; OA: osteoarthritis

Inclusion criteria	Exclusion criteria
18-75 years of age	Active local or systemic infection requiring antiviral, antibacterial, or antifungal treatment
Lateral hip pain >3 months' duration	BMI >40 kg/m²
History and clinical examination consistent with gluteal tendinopathy	Medical metabolic conditions, such as cancer, that might affect outcomes
MRI or sonographic evidence of gluteal tendinopathy	Chronic conditions such as CRPS or fibromyalgia that might affect pain symptoms
≥3 months of nonoperative treatment that included any of the following: nonsteroidal anti-inflammatory drugs; activity modification; physical therapy; previous steroid injections; prolotherapy; PRP, stem cell, or biologic injections; needle tenotomies; and other non-surgical treatments (e.g., shockwave, ultrasound, alternative minimally invasive tenotomy devices)	Current use of chronic pain medications for other conditions
	Grade III/IV OA changes in the hip joint or other conditions such as labral tear, femoroacetabular impingement, and iliopsoas bursitis
	Documented or suspected diagnosis of lumbar nerve root impingement, radiculopathy, etc.
	Previous surgery, including tendon and fracture fixation and joint arthroplasty
	Full-thickness tendon tears demonstrated radiologically
	Steroid injections, prolotherapy, and PRP, stem cell, or biologic injections within 12 weeks/3 months of the study procedure date
Willingness and ability to provide informed consent and comply with the study protocol	Needle tenotomy or alternative minimally invasive device interventions within 24 weeks/6 months of the study procedure date

Prospective patients meeting all the inclusion/exclusion criteria were asked to sign the study-specific IRB-approved informed consent form prior to enrollment and any study-specific tests or procedures. Once informed consent was obtained, baseline data (see Table 2) were collected during the patient enrollment visit.

**Table 2:** Baseline data collection during patient enrollment VAS: Visual Analog Scale; VISA-G: Victorian Institute of Sport Assessment-Gluteal Questionnaire; FABER: flexion-abduction-external rotation; FADIR: flexion-adduction-internal rotation

Baseline data
Demographic information: gender, age, weight, and height
Medical/surgical history; previous conservative treatment(s); current status
VAS pain score [18]
VISA-G [19]
FABER test
FADIR test
Modified Trendelenburg test
Physical exam
Palpation and inspection
Point tenderness at the lateral hip
Provocative tests (the following maneuver exacerbates lateral hip pain)
Hip abduction
Diagnostic imaging history
Pain medication usage

The primary endpoint of this registry was the reduction in pain as measured by the Visual Analog Scale (VAS). Secondary endpoints specific to gluteal tendinopathy included changes in Victorian Institute of Sport Assessment-Gluteal Questionnaire (VISA-G) scores, additional intervention(s), complications or adverse events related to the procedure and/or TenJet device, changes in medications following the procedure, and compliance with the post-procedure rehabilitation program.

Sonographic assessment of tendon pathology

Diagnostic ultrasound was performed to assess baseline tendon characteristics and the severity of tendon changes at the time of the index procedure using standardized grayscale and Doppler hyperemia criteria. Grayscale appearance was graded on a four-point scale ranging from 0 to 3, with 0 indicating normal tendon appearance, 1 representing mild hypoechogenicity, 2 indicating moderate diffuse hypoechogenicity with possible tendon swelling, and 3 reflecting severe changes characterized by anechoic clefts consistent with interstitial tearing. Hyperemia was also graded on a four-point scale ranging from 0 to 3, where 0 indicated no detectable flow, 1 represented mild scattered Doppler pixels, 2 corresponded to moderate linear or confluent flow involving less than 50% of the hypoechoic region, and 3 represented severe hyperemia involving greater than 50% of the affected tendon area. Sonographic grading was performed by the treating physician at each participating center, and no central blinded review or inter-rater reliability assessment was performed.

Index procedure

During the index outpatient procedure, the region of tendinopathy was identified by ultrasound and the site prepped using sterile techniques. Site-specific protocols were utilized for anesthesia and local anesthetic administration. Operation of the TenJet device was performed according to the manufacturer's instructions for use. During treatment, the working tip of the TenJet device was guided by the treating physician using continuous ultrasonography to perform targeted tenotomy and the resection of the degenerative tendon. Procedures were performed by physicians experienced in ultrasound-guided musculoskeletal interventions, with a mean of 4.9 years of experience utilizing TenJet (range: 2-9 years). Procedural parameters such as the number of passes, saline volume, or treatment time were individualized, as treating physicians had discretion to maneuver the device under continuous ultrasound visualization and treat the tendon until satisfactory changes were observed. Data collected during the index TenJet procedure are provided in Table 3.

**Table 3:** Data collected during the index TenJet procedure

Data
Treated tendon
Site of care (i.e., operating room, surgery center, etc.)
Procedure time/operating room time
Grading of tendon pathology based on ultrasound imaging
Type of anesthetic administered
Device malfunctions
Procedure complications/adverse events

Post-procedure protocol

Patients were discharged the same day when clinically stable. Prior to discharge, the investigator reviewed the post-procedure protocol for wound care, rehabilitation, and follow-up visits. All patients were required to return to the investigator's clinic at two weeks post-procedure to assess wound healing and to determine if the patient could initiate the rehabilitation exercise program. Patients were referred to a formal physical therapy program or provided home exercise guidelines at the discretion of the physician. This site-level discretion was intentional to reflect real-world differences in post-procedure rehabilitation across participating sites.

Subsequent follow-up clinic visits were scheduled for six weeks and/or three months, according to each site's standard-of-care protocol.

Patient-reported outcome surveys were emailed to the patient via the REDCap Cloud platform at the two-week, six-week, three-month, and six-month time points. Patients were contacted within the follow-up window via further emails or phone calls to urge them to complete the surveys.

Statistical analysis

Descriptive statistics were performed for all baseline characteristics, prior conservative treatments, procedural variables, and follow-up outcomes. Continuous variables are presented as mean±standard deviation and categorical variables as count (percentage). Normality of continuous data was assessed using the Shapiro-Wilk and Kolmogorov-Smirnov tests. Parametric data were compared using analysis of variance (ANOVA), while nonparametric data were compared using the Kruskal-Wallis test. Categorical variables were analyzed using the chi-squared or Fisher's exact tests as appropriate.

For longitudinal outcomes, an overall p-value assessing temporal differences across all time points was calculated, followed by baseline-referenced comparisons between each follow-up interval and the preoperative value using paired t-tests. No adjustment for multiple comparisons was performed for these pairwise analyses. Analyses were conducted using available case methodology without imputation. Statistical significance was defined as a two-tailed p-value of <0.05. All analyses were performed using R (R Foundation for Statistical Computing, Vienna, Austria).

## Results

Patient demographics and baseline characteristics

A total of 46 patients met the inclusion/exclusion criteria for gluteal tendinopathy treated with the TenJet system. The cohort was predominantly female (44/46, 95.6%) with a mean age of 60.7±8.44 years and a mean BMI of 26.9±4.73 kg/m². Laterality was evenly distributed, with 26 procedures (56.5%) performed on the left hip and 20 (43.5%) on the right. Diabetes and anticoagulant use were each reported in 2/46 (4.4%) of patients, while 46/46 (100%) identified as non-smokers (Table 4).

**Table 4:** Patient demographics

Demographics	Total (46)
Sex	
Female	44 (95.6%)
Male	2 (4.4%)
Age	60.7 (8.44)
BMI	26.9 (4.73)
Diabetes	2 (4.4%)
Anticoagulants	2 (4.4%)
Smoking: no	46 (100%)
Affected hip location	
Left	26 (56.5%)
Right	20 (43.5%)
Average duration of symptoms	44.1 months

Etiology of injury was most commonly idiopathic or degenerative, with 6/46 (13%) reporting an athletic cause of injury and 12/46 (26.1%) reporting a recreational cause, while 28/46 (60.9%) reported unknown onset. The duration of symptoms prior to the procedure ranged from three to 348 months, with a mean of 44.1±74.9 months (Table 5).

**Table 5:** Mechanism of injury

Cause of injury	Total (46)
Recreation	12 (26.1%)
Athletics	6 (13%)
Work	0 (0%)
Unknown	28 (60.9%)

Prior to being evaluated by the treating physician, 27/46 (58.7%) of patients had undergone one MRI evaluation (mean: 0.72±0.72 MRI; range: 0-4), and 28/46 (60.1%) had received at least one diagnostic musculoskeletal ultrasound evaluation (mean: 0.76±0.77 ultrasound; range: 0-3) (Table 6).

**Table 6:** Patient-reported prior interventions and diagnostic imaging (n=46)

Prior interventions and diagnostic imaging	n (reported)	%
Prior corticosteroid injections (avg=1.97 (2.61))		
1	17	36.9%
2	9	19.5%
3+	11	23.9%
Total	37	80.4%
Prior formal physical therapy sessions (avg=5.06 (7.43))		
<2	5	10.8%
5-6	5	10.8%
8	2	4.3%
10	3	6.5%
12	4	8.7%
17+	5	10.9%
Total	24	52.2%
Prior MRI visits (avg=0.72 (0.72))		
1	27	58.7%
2	1	2.2%
3	1	2.2%
Total	29	63%
Prior ultrasound visits (avg=0.76 (0.77))		
1	23	50%
2	3	6.5%
3	2	4.3%
Total	28	60.1%

Prior to the index procedure, all patients had failed at least one form of conservative treatment. Corticosteroid injections were frequently used, with 17/46 (36.9%) of the patients receiving one injection and 20/46 (43.4%) receiving two or more injections (mean: 1.97±2.61; range: 0-15). Physical therapy was utilized by 24/46 (52.2%) of patients. Five out of 46 (10.8%) patients completed fewer than two sessions, 14/46 (30.4%) attended between five and 12 sessions, and 5/46 (10.9%) exceeded 17 sessions (mean: 5.06±7.43; range: 0-30) (Table 6). Over-the-counter (OTC) pain medication use was reported by 35/46 (76.1%) preoperatively, and prescription pain medication use was reported by 6/46 (13%) preoperatively (Table 7).

**Table 7:** Patient-reported prior medication use (n=46)

Prior pain medications	n (reported)	%
Over-the-counter	35	76.1%
Prescription	6	13%
Total	41	89.1%

Procedural characteristics and intraoperative ultrasound findings

Procedures were performed predominantly in the outpatient setting, with 27/46 (58.7%) conducted in a hospital or clinic procedure room, 14/46 (30.4%) in a surgery center, and 5/46 (10.9%) in a hospital operating room. Twelve out of 42 (28.6%) cases were completed within 30 minutes, 21/42 (50%) between 30 and 45 minutes, and 9/42 (21.4%) between 45 and 60 minutes. Room time data were available for 42/46 (91.3%) procedures. Local anesthetic was administered in 45/46 (97.8%) of cases. No device malfunctions or immediate intra- or post-procedural complications were recorded (Table 8).

**Table 8:** Site of care, procedural characteristics, and intraoperative imaging MAC: monitored anesthesia care

	n (reported)	%
Site of care		
Hospital, procedure room	10	21.7%
Hospital operating room	5	10.9%
Surgery center	14	30.4%
Clinic procedure room	17	37%
Total	46	100%
Room time		
30 minutes	12	28.6%
45 minutes	21	50%
60 minutes	9	21.4%
Total	42	91.3%
Anesthetic		
Local only	45	97.8%
MAC and local	0	0%
Total	45	97.8%
Device malfunctions	0	0%
Complications	0	0%
Ultrasonographic grading		
Grayscale, normal	0	0%
Grayscale, mild	8	18.2%
Grayscale, moderate	30	68.2%
Grayscale, severe	6	13.6%
Total	44	95.6%
Hyperemia, no flow	34	75.6%
Hyperemia, mild	8	17.8%
Hyperemia, moderate	3	6.7%
Hyperemia, severe	0	0%
Calcifications	5	11.1%
Total	45	97.8%

Sonographic grading at the time of treatment, assessed manually by the operating physician using the grayscale, demonstrated mild hypoechogenicity in 8/44 (18.2%), moderate in 30/44 (68.2%), and severe tendinopathy/interstitial tearing in 6/44 (13.6%) of cases. Grayscale sonographic grading was available for 44/46 (95.6%) procedures. Hyperemia, as assessed by the treating physician, showed detectable Doppler flow in 11/45 (24.4%) of tendons, and calcifications were identified in 5/45 (11.1%). Doppler flow and calcification assessments were available for 45/46 (97.8%) procedures (Table 8).

Post-procedural wound healing, medication utilization, and rehabilitation

Incision healing progressed as expected, with the majority of incisions healed by two weeks and all incisions healed by three months (overall p=0.005). 

OTC pain medication use was reported in 35/46 (76.1%) preoperatively and decreased significantly over time to 20/46 (43.5%) at two weeks, 11/46 (23.9%) at six weeks, and 6/44 (13.6%) at three months (overall p<0.001). Prescription pain medication use was less common and did not demonstrate a significant change across follow-up time points (Table 9).

**Table 9:** Post-procedural utilization of pain medication

Time point	n (reported)	None	%	Over-the-counter	%	Prescription	%
2 weeks	46	18	39.1%	20	43.5%	8	17.4%
6 weeks	46	31	67.4%	11	23.9%	4	8.7%
3 months	44	38	86.4%	6	13.6%	2	4.5%

Of those patients who reported formal post-procedural physical therapy participation or a home exercise program, compliance remained high throughout follow-up, with the majority of patients participating in either formal physical therapy or a home exercise program at two weeks, six weeks, and three months (Table 10).

**Table 10:** Adherence to post-procedural rehabilitation/home exercise program

Time point	n (reported)	None	%	Formal physical therapy	%	Home exercise program	%
2 weeks	41	4	9.8%	13	31.7%	24	58.5%
6 weeks	37	4	10.8%	16	43.2%	17	45.9%
3 months	18	5	27.8%	9	50%	4	22.2%

The patient-reported outcome measure survey completion rates at each follow-up interval are summarized in Table 11.

**Table 11:** Patient-reported outcome measure survey responses by time point Forty-three patients completed at least one VAS or VISA-G survey at six months. VAS: Visual Analog Scale; VISA-G: Victorian Institute of Sport Assessment-Gluteal Questionnaire

Total n=46	Pre-op	2 weeks	6 weeks	3 months	6 months
VAS	43 (93.5%)	43 (93.5%)	42 (91.3%)	42 (91.3%)	40 (87%)
VISA-G	43 (93.5%)	40 (87%)	43 (93.5%)	36 (78.3%)	36 (78.3%)

At baseline, the mean VAS pain score was 5.81±2.02 (n=43). Significant improvement was observed by two weeks (3.14±2.24; p<0.001) and continued through all follow-up intervals, with mean scores of 2.40±2.00 at six weeks (p<0.001), 2.17±1.87 at three months (p<0.001), and 2.10±2.10 at six months (p<0.001) (Table 12). Thirteen out of 43 (30.2%) patients were nearly pain-free with a VAS score of 0-1 at two weeks. Eighteen out of 42 (42.9%) patients had a VAS of 0-1 at three months and 20/40 (50%) at six months.

**Table 12:** Visual Analog Scale scores over time The table shows the changes in reported pain levels from preoperative through the six-month follow-up. Follow-up p-values are relative to preoperative baseline; the overall p-value reflects change across time points.

	Pre-op (N=43)	2 weeks (N=43)	6 weeks (N=42)	3 months (N=42)	6 months (N=40)	P-value	F statistic
Visual Analog Scale	5.81 (2.02)	3.14 (2.24)	2.40 (2.00)	2.17 (1.87)	2.10 (2.10)	<0.001	F=24.1
P-value	-	<0.001	<0.001	<0.001	<0.001	-	-

VISA-G scores improved from 44.9±14.3 at baseline (n=43) to 49.0±19.1 at two weeks (p=0.807), 62.2±15.8 at six weeks (p<0.001), 66.9±15.2 at three months (p<0.001), and 69.6±12.6 at six months (p<0.001) (Table 13).

**Table 13:** Victorian Institute of Sport Assessment-Gluteal Questionnaire scores over time The table demonstrates the functional recovery and patient-reported outcome trends through the six-month follow-up. Follow-up p-values are relative to preoperative baseline; the overall p-value reflects change across time points.

	Pre-op (N=43)	2 weeks (N=40)	6 weeks (N=43)	3 months (N=36)	6 months (N=36)	P-value	F statistic
VISA-G	44.9 (14.3)	49.0 (19.1)	62.2 (15.8)	66.9 (15.2)	69.6 (12.6)	<0.001	F=19.68
P-value	-	0.807	<0.001	<0.001	<0.001	-	-

Additional interventions and complications

A limited number of patients required adjunctive management during follow-up. At two weeks, one patient was prescribed a prednisone taper, and one patient developed a localized adhesive-related rash without further issues. At six weeks, the previously mentioned patient completed the prednisone taper, and a distinct patient began a prescription course of the nonsteroidal anti-inflammatory drug (NSAID) celecoxib (Celebrex). No complications were reported at this interval. By two months, the patient prescribed celecoxib had discontinued the NSAID and home exercise program and underwent a left lateral gluteus medius corticosteroid injection. No additional complications were documented.

## Discussion

Gluteal tendinopathy represents a spectrum of pathology involving the abductor tendons of the lateral hip ranging from inflammation, degeneration, and tearing. Historically, treatments have been geared towards addressing inflammation rather than underlying degenerative changes that occur within the tendon associated with tendinosis. Typically, management of the condition is started with conservative options such as activity modifications, NSAIDs, corticosteroid injection, and potentially physical therapy. Prior literature describes that 78% were improved with exercise and physiotherapy at 52 weeks compared to 57% who received a corticosteroid injection and 52% with a wait-and-see approach [11]. For those who remain symptomatic after conservative treatment, there is a lack of consensus regarding the next step in management. PRP, ultrasound-guided tenotomy, extracorporeal shockwave therapy, and surgical repair have all been proposed as options for recalcitrant cases [20-26]. Although prior studies have reported favorable outcomes following percutaneous tenotomy techniques for gluteal tendinopathy and GTPS, prospective multicenter data evaluating ultrasound-guided hydroresection of pathologic gluteal tendon tissue remain limited [27-31]. This interim analysis provides the real-world framework and insights into clinical outcomes through a six-month follow-up after the ultrasound-guided treatment of chronic refractory gluteal tendinopathy with the TenJet device.

Our data demonstrated that treatment with TenJet was associated with significant improvements in pain and patient-reported function at six months (p<0.001) in individuals with recalcitrant gluteal tendinopathy. The mean VAS pain scores improved from 5.8 pre-procedure to 2.1 at six months, representing a reduction of approximately 3.7 points. This value exceeds the minimal clinically important difference (MCID) of a 1.48-point reduction in the Numeric Rating Scale (NRS) which has been widely accepted as clinically meaningful in chronic musculoskeletal pain populations [32]. Our cohort maintained MCID thresholds for numeric pain at all time points from two weeks to six months, indicating clinically meaningful pain reduction early after treatment with sustained benefit through follow-up. VISA-G scores showed marked improvement over time, increasing from 44.9 at baseline to 69.6 at six months.

These findings indicate improvements in patient-reported function following treatment. These improvements were supported by statistically significant reductions in pain and functional limitations. VAS scores improved significantly at all postoperative time points, while VISA-G scores demonstrated significant gains beginning at six weeks (p<0.001), which were sustained at three months (p<0.001) and six months (p<0.001). Collectively, these data suggest that TenJet was associated with early and sustained improvements in pain and patient-reported physical function within the first six months following intervention.

The smallest detectable change for VISA-G is 18.4 [33]. Our cohort exceeded that change with documented improvement of 24.7 at six months. Although the proposed VISA-G MCID of 29 was not reached, this threshold was derived from open surgical cohorts with more severe pathology of the gluteal tendon with partial- and full-thickness tears and higher expectations for recovery. This may increase the magnitude of change required to define MCID when compared to open surgical outcomes. In contrast, our cohort underwent a minimally invasive intervention with a different disease population, with a mean improvement of 24.7 points, which exceeded the reported smallest detectable change of 18.4 points but did not reach the proposed MCID of 29 points. The investigators look forward to the longer-term data to see if MCID is achieved at 12 months and 24 months post-procedure.

The mechanism of action of TenJet may contribute to these favorable outcomes. Unlike conservative treatments, TenJet utilizes a high-pressure saline jet to selectively resect pathologic tendon tissue while preserving healthy fibers [24,25]. This approach facilitates the removal of degenerative tissue and promotes a regenerative healing response, including collagen realignment. This mechanism parallels surgical debridement techniques used in open or arthroscopic procedures. Given that chronic gluteal tendinopathy is characterized by a failed healing response rather than active inflammation, targeted removal of the diseased tendon matrix is likely central to symptom reduction and functional recovery [34,35].

Disease severity is not predictive of outcomes when treating common extensor tendinopathy of the lateral elbow with TenJet. However, this may not apply to treatment of the gluteal tendons [24,25]. In this study, 36/44 (81.8%) of the cohort had moderate or severe tendinosis based on ultrasound grading. Five out of 45 (11.1%) of the cohort had concurrent calcifications, and another 13.6% had partial tendon tearing. This indicates that a large portion of the cohort had advanced and severe disease. Future studies, if powered appropriately, may be able to detect differences in outcomes based on the severity of tendon disease. 

Importantly, no device malfunctions, infections, or major adverse events were reported in this cohort, supporting the safety of the procedure. This is similar and consistent with other studies utilizing TenJet [24,25]. Taken together, these findings suggest that ultrasound-guided minimally invasive tenotomy with TenJet was associated with a favorable short-term safety profile and improvements in pain and function for patients with refractory gluteal tendinopathy who have failed conservative management.

This study has multiple strengths due to the prospective, multicenter study design. No other study has prospectively evaluated patient outcomes after treatment using TenJet's novel mechanism of hydroresection for recalcitrant gluteal tendinopathy. The multicenter study design evaluates results from a diverse group of physicians and may extend generalizability to other physicians who perform these procedures. Additional strengths include capturing ultrasound findings, capturing intraprocedural data, and utilizing validated patient-reported outcome measures, including VAS and VISA-G. Furthermore, no patients required reoperation or surgical intervention. Only one patient underwent further intervention with a subsequent corticosteroid injection due to acute inflammation not managed by NSAIDs or oral prednisone.

This study has several limitations. First, the study design was a single-arm registry without a comparison group, limiting causal interpretation because observed improvements may also reflect natural history, regression to the mean, or postprocedural rehabilitation and other treatments. Second, the sample size was modest, which may limit the generalizability of findings or further detailed analysis of certain clinical factors such as stages of tendinopathy or duration of symptoms. Although sonographic grading was performed by treating physicians experienced in musculoskeletal ultrasound, grading was performed without a central reading protocol, blinded review, or assessment of inter-rater reliability. The exclusion of patients with full-thickness tears, advanced osteoarthritis, and prior hip surgery may also limit generalizability to patients with more complex pathology. The cohort was predominantly female (44/46, 95.6%), which may limit generalizability to males. However, prior literature has reported GTPS as affecting females: males at rates as high as 9:1 [3,13,31]. Three patients were lost to follow-up after three months. Response rates for some secondary measures were also limited, particularly rehabilitation adherence at three months (18/46), which may limit the interpretation of these findings. Although VISA-G improved at six months, the mean change did not reach the previously proposed MCID of 29 points, which should be considered when interpreting the functional improvement. The present interim analysis is limited to six months, representing short-term improvements in symptoms and function. Despite these limitations, this registry provides real-world clinical data demonstrating significant improvements in pain and patient-reported function following treatment. Similar outcome measures will be collected for this cohort at 12 and 24 months and will be reported after study completion.

## Conclusions

At the six-month follow-up, this interim analysis of a prospective, single-arm study demonstrated significant pain reduction and functional improvement following treatment with the TenJet system for recalcitrant gluteal tendinopathy. TenJet was associated with a favorable short-term safety profile, with no device malfunctions, infections, or device-related adverse events reported. This ultrasound-guided tenotomy and resection technique represents an alternative treatment option for patients who have failed conservative management, bridging the gap between nonoperative care and surgical intervention. Publication of the results at longer-term follow-up and additional prospective, controlled studies are warranted to further validate these findings and further define TenJet's role in the treatment algorithm for GTPS.
